# Muscle Expression of SOD1^G93A^ Modulates microRNA and mRNA Transcription Pattern Associated with the Myelination Process in the Spinal Cord of Transgenic Mice

**DOI:** 10.3389/fncel.2015.00463

**Published:** 2015-12-01

**Authors:** Gabriella Dobrowolny, Camilla Bernardini, Martina Martini, Mirko Baranzini, Marta Barba, Antonio Musarò

**Affiliations:** ^1^DAHFMO-Unit of Histology and Medical Embryology, Institute Pasteur-Cenci Bolognetti, IIM, Sapienza University of RomeRome, Italy; ^2^Center for Life Nano Science at Sapienza, Istituto Italiano di TecnologiaRome, Italy; ^3^Institute of Anatomy and Cell Biology, Università Cattolica del Sacro CuoreRome, Italy

**Keywords:** muscle-nerve interplay, miRNA and mRNA signature, ALS, myelination process, SOD1^G93A^

## Abstract

A crucial system severely affected in several neuromuscular diseases is the loss of effective connection between muscle and nerve, leading to a pathological non-communication between the two tissues. One of the best examples of impaired interplay between muscle and nerve is Amyotrophic Lateral Sclerosis, a neurodegenerative disease characterized by degeneration of motor neurons and muscle atrophy. Increasing evidences suggest that damage to motor neurons is enhanced by alterations in the neighboring non-neuronal cells and indicate that altered skeletal muscle might be the source of signals that impinge motor neuron activity and survival. Here we investigated whether muscle selective expression of SOD1^G93A^ mutant gene modulates mRNAs and miRNAs expression at the level of spinal cord of MLC/SOD1^G93A^ mice. Using a Taqman array, the Affymetrix Mouse Gene 2.0 ST approach and the MiRwalk 2.0 database, which provides information on miRNA and their predicted target genes, we revealed that muscle specific expression of SOD1^G93A^ modulates relevant molecules of the genetic and epigenetic circuitry of myelin homeostasis in spinal cord of transgenic mice. Our study provides insights into the pathophysiological interplay between muscle and nerve and supports the hypothesis that muscle is a source of signals that can either positively or negatively affect the nervous system.

## Introduction

Amyotrophic Lateral Sclerosis (ALS) is a fatal neurodegenerative disorder in which the functional connection between nerve and muscle is severely compromised (Musarò, 2013). ALS affects pyramidal neurons in the motor cortex and lower motor neurons originating in the brainstem and in the spinal cord. The most typical feature of this progressive lethal disease is the degeneration of motor neurons, muscle weakness and atrophy, that leads to progressive paralysis and death by respiratory failure in few years (Musarò, 2010, 2013). ALS is epidemiologically classified into two major forms: sporadic (90–95%) and familial (5–10%) form. Among the familial cases, ~20% are caused by dominantly inherited mutations in the Cu/Zn superoxide dismutase (SOD1) protein (Rosen, 1993) a cell scavenger of superoxide.

One of the critical issue that remains to be addressed in ALS is whether motor neurons are the first and sole direct targets of ALS or if the toxic activity of mutant SOD1 is cell autonomous. Recent experimental evidence revealed that ALS is a multi-factorial and multi-systemic disease in which alterations in structural, physiological, and metabolic parameters in different cell types, such as motor neurons, glia, and muscle cells, may act synergistically to exacerbate the disease (Boillée et al., 2006; Julien, 2007; Musarò, 2013). Skeletal muscle is a source of signals that influence neuron survival, axonal growth and maintenance of synaptic connections. Notably, development in the absence of skeletal muscle results in the sequential ablation of motor neurons from the spinal cord to the brain (Kablar and Rudnicki, 1999); thus, effective connection between muscle and nerve is crucial for the survival and function of both tissues throughout life. We have previously demonstrated that muscle-specific expression of SOD1 mutation in the MLC/SOD1^G93A^ transgenic mouse model, which selectively expresses the mutant SOD1^G93A^ gene in the skeletal muscle under the transcriptional control of a muscle-specific promoter (MLC), induces accumulation of Reactive Oxygen Species, causes muscle atrophy with a concomitant alteration in the ultrastructure and in the functional performance of skeletal muscles, and promotes microglia activation in the spinal cord, that is a presymptomatic sign of ALS disease (Dobrowolny et al., 2008b). Moreover, it has been reported that skeletal muscle-restricted expression of the human mutant SOD1 gene causes motor neuron degeneration in old transgenic mice (Wong and Martin, 2010) and that muscle-selective alterations in mitochondrial function initiate neuromuscular junction alteration, distal axonopathy, astrocytosis, and mild motor neuron loss (Dupuis et al., 2009). We also demonstrated that forced expression of muscle specific isoform of the Insulin Growth Factor-1 (mIGF-1) exclusively in the skeletal muscle of the global SOD1^G93A^ mice counteracted the symptoms of ALS, induced satellite cell activation, stabilized neuromuscular junctions, preserved motor peripheral nerve, and led to a reduction in astrocytosis in the SOD1^G93A^ spinal cord (Dobrowolny et al., 2005, 2008a). All together these data indicate that nervous development and homeostasis are intimately coupled to skeletal myogenesis and muscle function. Moreover, these findings support the hypothesis that skeletal muscle is a primary target of mutant SOD1 toxicity in mice and indicate that altered skeletal muscle impairs motor neuron activity, suggesting a sequential pattern of degeneration in which muscle abnormalities precede motor neuron death. In order to add new insights into the pathogenesis of ALS and define whether skeletal muscle alteration potentially affect the nervous system we analyzed the mRNA and miRNA expression in the lumbar ventral spinal cord of MLC/SOD1^G93A^ transgenic mouse model (Dobrowolny et al., 2008b). Interestingly, we found that muscle specific expression of SOD1^G93A^ gene affects spinal cord miRNome and transcriptome; in particular we observed the activation of genes involved in the myelination process and a decrease in the axon diameter to total fiber diameter in the sciatic nerve, suggesting a myelinopathy in the transgenic mouse model.

## Materials and methods

### Mice

Animal model used: 4 month old MLC/SOD1^G93A^ mice overexpressing the mutant SOD1 gene (SOD1^G93A^) under the control of the Myosin Light Chain (MLC) muscle specific promoter (Dobrowolny et al., 2008b) and 4 month old FVB (Friend leukemia virus B) (control strain). The animals were housed in a temperature-controlled (22°C) room with a 12:12 h light-dark cycle. All animal experiments were approved by the ethics committee of the Unit of Histology and Medical Embryology-Sapienza University of Rome- and were performed in accordance with the current version of the Italian Law on the Protection of Animals.

### RNA isolation

Total RNA was isolated from frozen lumbar spinal ventral tissue specimens using pestel homogenization, TRIzol reaction (Invitrogen, Carlsbad, CA, USA), and further on-column purification as previously described. The yield, quality and integrity of RNA were determined using the Agilent 2100 Bioanalyzer (Agilent Technologies, Palo Alto, CA, USA) as previously described (Bernardini et al., 2012).

### miRNA analysis

From each sample (*n* = 4 per experimental group), 600 ng of total RNA was reverse transcribed using Taqman MicroRNA Reverse Transcription Kit with Megaplex Primer Pools A & B (Applied Biosystems, Foster City, CA, USA). The cDNA was analyzed with Taqman Array Mouse MicroRNA A and B Cards Set version 3 (Applied Biosystems, Foster City, CA, USA) on the Applied Biosystems 7900HT qPCR system (Applied Biosystems, Foster City, CA, USA). Most of the MicroRNAs in the B Card were undetermined, therefore their analysis was omitted. The data was normalized by global mean normalization. SDS RQ Manager version 2.4 (Applied Biosystems, Foster City, CA, USA) was used to calculate Ct values (number of cycles required for the fluorescent signal to cross the set threshold), Ct values greater than 35 were considered as non-specific or undetected, and were filtered. MicroRNAs with missing values in at least 2 of 4 replicate samples per treatment group were removed from the dataset. The remaining Ct values were normalized using the U6 reference miRNA as suggested by manufacturer's protocol. The fold change between sample groups was calculated by subtracting the relative expression of the samples and then transforming the result to anti-log2 (i.e., 2^difference^).

Relevant information about the experiment, is available at http://www.ncbi.nlm.nih.gov/geo/ under accession GSE71193.

### miRNA pathway analysis

The putative correlation between mRNAs and miRNAs was investigated through the MiRwalk 2.0, a comprehensive database that provides information on miRNA from Human, Mouse and Rat on their predicted as well as validated binding sites on their target genes. In particular we get information on miRNA produced by 8 established miRNA prediction programs on 3′ UTRs of all known genes of Mouse (Dweep et al., 2011; Bernardini et al., 2012).

miRNA predicted pathways were identified using DIANA (DNA Intelligence Analysis) tools (Vlachos et al., 2012).

DIANA-miRPath is a miRNA pathway analysis web-server that utilizes predicted miRNA targets (in CDS or 3′-UTR regions) provided by the DIANA-microT-CDS algorithm or even experimentally validated miRNA interactions derived from DIANA-TarBase v6.0. These interactions (predicted and/or validated) can be subsequently combined with sophisticated merging and meta-analysis algorithms. Essentially, this tool allows identifying all the predicted or experimentally validated miRNAs significantly targeting a selected pathway.

### Single qPCR validation

Single qPCR of miRNA 133a, 133b, 9, 29, 330, and 1 was performed to validate the performance of the Taqman arrays using Taqman probes from single tube Taqman microRNA assays with the diluted cDNA in a 10 μl reaction volume, in triplicate for each assay, on the Applied Biosystems 7500HT system (Applied Biosystems, Foster City, CA, USA; *n* = 10 per experimental group).

### Western blot analysis

Protein extraction from both wild type and MLC/SOD1^G93A^ transgenic spinal cord (*n* = 5 for each genotype) was performed in 10 mM Tris, 150 mM Sodium Chloride, 1% NP40, 0.1% SDS, 10% Glycerol, 1% Deoxycholate, 1 mM Phenylmethylsulfonyl Fluoride, 1 μg/ml Aprotinin, 1 μg/ml Leupeptin, 1 μg/ml Pepstatin, 1 mM Sodium Orthovanadate, and 1 mM Sodium Fluoride. Equal amounts of protein from each lysate were separated in SDS polyacrilamide gel and transferred onto a nitrocellulose membrane. Filters were blotted with antibodies against Pmp22 (Novus Biological Littleton, CO, USA. Cat. NBP1-67670), Mpz (Abcam, Cambridge, UK. Cat. Ab64685), and a-tubulin (Sigma, Saint Louis, MO, USA. Cat. T5168).

Pmp22 primary antibody was diluted 1:200 in TBST and used for blotting over night (o.n.) after 5% milk saturation. Then, filter was incubated with secondary antibody (Goat anti-Rabbit IgG HRP-conjugated) (Bethyl, Montgomery, TX, USA. Cat. A120-201P) in 1% milk for 1 h.

Mpz primary antibody was diluted 1:500 in TBST and used for blotting o.n. after 10% milk saturation. Then, filter was incubated with secondary antibody (Goat anti-Mouse IgG HRP-conjugated) (Bethyl, Montgomery, TX, USA. Cat. A90-516P) in 1% milk for 1 h.

Tubulin was diluted 1:5000 in TBST and used for blotting o.n. after 5% milk saturation. Then, filter was incubated with secondary antibody (Goat anti-Mouse IgG HRP-conjugated) (Bethyl Montgomery, TX, USA. Cat. A90-516P) in 1% milk for 1 h. Signals were acquired by ChemiDoc MP instrument (Bio-Rad, Hercules, CA, USA) and processed with Image Lab acquisition analysis software (Bio-Rad, Hercules, CA, USA).

Five independent samples for each group of animals have been used for densitometric analysis and protein level of α-tubulin was used as control for equal protein loading.

### Microarray analysis

Total RNA was used to create the biotin-labeled library to be hybridized on GeneChip® Mouse Gene 2.0 ST Array (Affymetrix, Santa Clara, CA, USA) covering more than 26500 RefSeq coding transcripts and more than 3500 RefSeq non-coding transcripts, following the manufacturer protocol.

Briefly, double-stranded cDNA was synthesized routinely from less than 1 microgram of total RNA primed with a poly-(dT)—T7 oligonucleotide. The cDNA was used in an *in vitro* transcription reaction in the presence of T7 RNA polymerase and biotin-labeled modified nucleotides during 16 h at 37°C. Biotinylated cRNA was purified and then fragmented (35–200 nucleotides), together with hybridization controls and hybridized to the microarrays for 16 h at 45°C. Using the GeneChip Fluidics Station 450 (Affymetrix, Santa Clara, CA, USA), the biotin-labeled cRNA was revealed by successive reactions with streptavidin R-phycoerythrin conjugate, biotinylated anti-streptavidin antibody and streptavidin R-phycoerythrin conjugate. The arrays were finally scanned in an Affymetrix GeneChip Scanner 7G Plus (Affymetrix, Santa Clara, CA, USA).

### Gene expression data analysis—differentially expressed gene list

The CEL files that store the results of the intensity calculations on the pixel values collected from an Affymetrix scanner and result from the hybridization, were analyzed using Transcriptome Analysis Console (TAC) from Affymetrix (Affymetrix, Santa Clara, CA, USA). Gene-level calculation was performed by Robust Multichip Average to summarize probeset signal (Irizarry et al., 2003) and normalization by quantile sketch (Bolstad et al., 2003). A data table (rma), together with the relative CEL and relevant information about the experiment, is available at http://www.ncbi.nlm.nih.gov/geo/ under accession GSE69582.

An unpaired One-way ANOVA was then used to identify differentially expressed genes, only the genes which met our criterion (*p* < 0.05, fold change > 1.5) were selected in this study. The resulting gene list was then annotated according to the Gene Ontology (GO) database (www.geneontology.org). This allowed assigning a category to each gene in the list, according to three defined “ontologies” (i.e., terms representing gene product properties): cellular component, biological process and molecular function.

The expression of selected genes was quantified in real time PCR to obtain an independent validation of microarray data. Real time PCR was carried out as previously described elsewhere (Bernardini et al., 2012).

### Functional categorization

The gene expression list has been functionally categorized using the Database for Annotation, Visualization, and Integrated Discovery (DAVID; http://david.abcc.ncifcrf.gov/) (Sherman et al., 2007). The algorithms implemented in this software allow identifying over-represented gene ontology (GO) terms with respect to the total number of genes assayed and annotated. To this aim, DAVID applies a modified Fisher exact test, to establish if the proportion of genes falling into an annotation category significantly differs from the background group of genes. In addition, this tool enables the fine mapping of genes within well-defined signaling and/or metabolic pathways, classified in the Kyoto Encyclopedia of Genes and Genomes (KEGG) database (www.genome.jp/kegg/). The KEGG mapping tool was employed for the functional categorization of the gene regulatory networks. For this purpose, AffyGene IDs, corresponding to the genes in the selected list, were used as queries and the whole set of genes represented on the array was used as the background group. A *p* < 0.05 was set.

The STRING (Search Tool for the Retrieval of Interacting Genes/Proteins) online software (Franceschini et al., 2013) was used to search interaction relationships of the proteins encoded by differential expressed genes.

### Histological analysis

Sciatic nerves were dissected and quickly fixed in 2.5% glutaraldehyde in 0.1 M sodium cacodylate for 2 h, washed in 0.1 M sodium cacodylate. Nerves were then post-fixed in 1% osmium in 0.1 M sodium cacodylate for 1 h, dehydrated in ethanol and embedded in epoxy resin. Samples were infiltrated with toluene and cut with a Leica ultracut R ultramicrotome (Leica Microsystems Inc. Buffalo Grove, IL) to obtain 2 um suitable for toluidine blue staining. Microscope using 40 × lenses was employed, and images were processed using Axiovision Rel 4.8 (Zeiss, Oberkochen, D) representative images of sciatic nerve thin sections are shown cropped in **Figure 5**.

The g-ratio was calculated as (dmax + dmin)/2/(Dmax + Dmin)/2 and the analysis was performed for 200 fibers. Each diameter was calculated from the average of the major axis (Dmax: maximum fiber diameter; dmax:maximum axonal diameter) and the minor axis (Dmin: minimum fiber diameter; dmin: minimum axonal diameter), (Tsutsumi et al., 2014) and was measured using Image J software (Image J 1.36, National Institutes of Health, MD, USA).

### Statistical analysis

Statistical analysis, if not differently specified was performed using the Mann Whitney nonparametric test and significance was established for *p* < 0.05.

## Results

### miRNA and mRNA expression profile of the spinal cord of the MLC/SOD1^G93A^ mice

**Figure 1:**
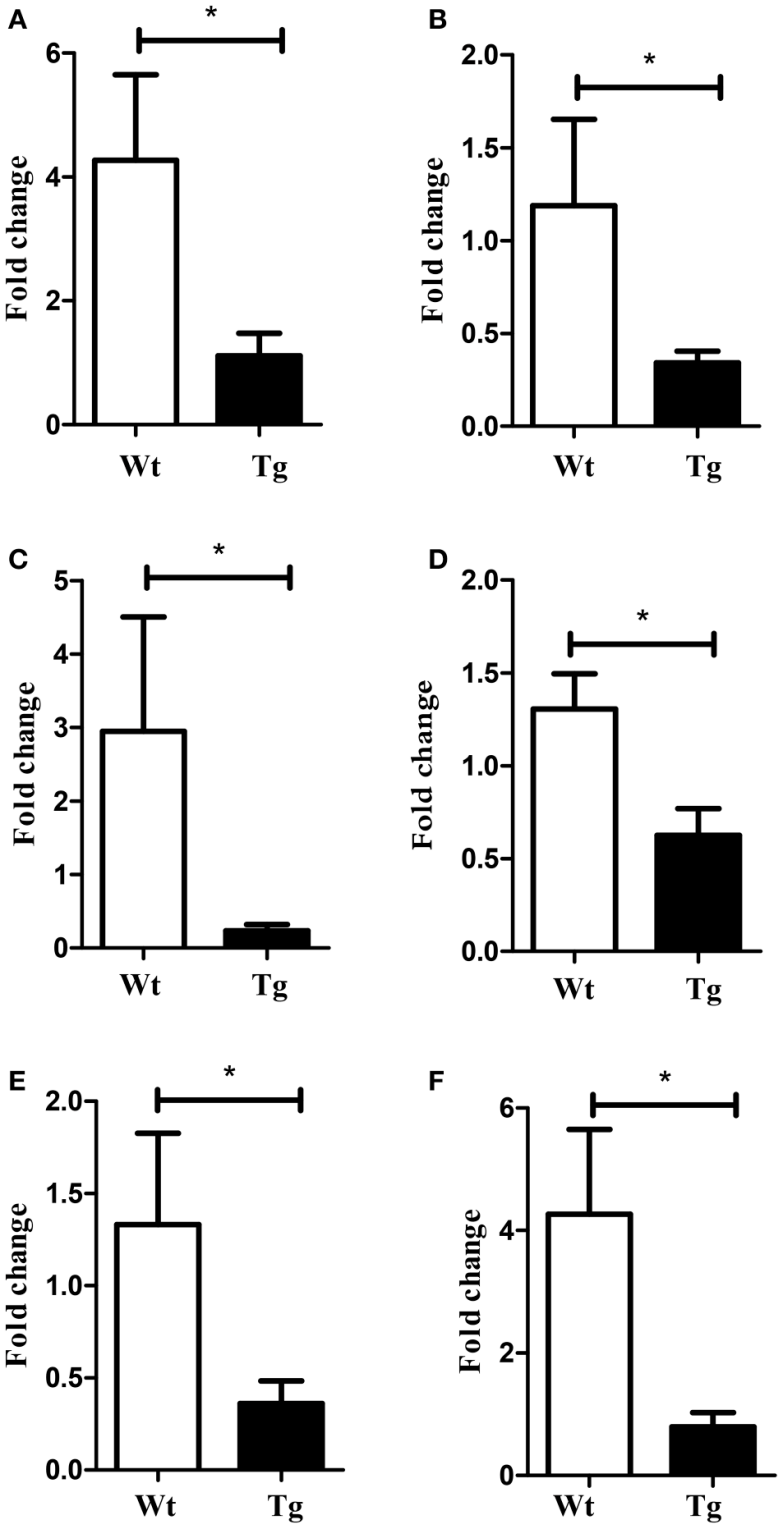
**Validation of selected miRNAs array data by Real-time PCR**. Graphs indicate relative expression of **(A)** mir-133a **(B)** mir-133b **(C)** mir-9 **(D)** mir-29 **(E)** mir-330 **(F)** mir-1. White bar refers to wild type (Wt) and black bar to MLC/SOD1^G93A^ (Tg). Values are expressed as mean ± SEM. ^*^p < 0.05 (*n* = 10 for each group).

First, to identify deregulated miRNAs and transcripts in the spinal cord of MLC/SOD1^G93A^ transgenic mice we performed miRNA expression profiling and DNA microarray, using the Taqman array card and Affymetrix Mouse Gene 2.0 ST respectively (GSE71194). Among the analyzed miRNAs, 54 were significantly down-regulated (*p* < 0.05) in the spinal cord of MLC/SOD1^G93A^ transgenic mice compared to wild type littermates (Table 1). Of note, the miRnome profiling revealed the down regulation of mir-330, mir-133, and mir-1, which are involved in denervation and reinnervation processes (Jeng et al., 2009; Tsutsumi et al., 2014). In addition mir-29 and mir-9 family member were downregulated and their deregulation was associated with different neurodegenerative diseases, including Huntington, Alzheimer, and Parkinson diseases (Saito and Saito, 2012; Tsutsumi et al., 2014) and demyelination-related diseases (Li and Yao, 2012; Tsutsumi et al., 2014). Real time PCR analysis was performed to validate the expression of these relevant miRNAs in the spinal cord of both wild type and MLC/SOD1^G93A^ transgenic mice (Figure 1).

**Table 1:** **List of significantly down-regulated miRNAs in MLC/SOD1^G93A^ spinal cord**.

**miRNA**	**Fold change**	***P*-value**
mmu-let-7b	−4.97	0.04170
mmu-let-7c	−4.80	0.01392
mmu-let-7d	−3.85	0.02516
mmu-let-7i	−3.92	0.04791
mmu-miR-100	−4.31	0.02747
mmu-miR-1	−3.69	0.01614
mmu-miR-106a	−5.23	0.02034
mmu-miR-106b	−4.57	0.03639
mmu-miR-10a	−4.07	0.01796
mmu-miR-10b	−3.75	0.03798
mmu-miR-128a	−4.69	0.02756
mmu-miR-129-3p	−3.55	0.03832
mmu-miR-129-5p	−7.58	0.00264
mmu-miR-133a	−2.75	0.03894
mmu-miR-133b	−3.94	0.00340
mmu-miR-15a	−8.16	0.00251
mmu-miR-15b	−3.84	0.02226
mmu-miR-17	−4.43	0.04014
mmu-miR-181a	−4.35	0.02206
mmu-miR-181c	−7.37	0.01568
mmu-miR-188-5p	−4.20	0.03755
mmu-miR-192	−3.38	0.04185
mmu-miR-194	−3.60	0.01944
mmu-miR-196b	−3.24	0.04461
mmu-miR-199a-3p	−4.67	0.01722
mmu-miR-19b	−5.56	0.03463
mmu-miR-20b	−6.42	0.02596
mmu-miR-23b	−4.66	0.04223
mmu-miR-26b	−5.25	0.01406
mmu-miR-29a	−4.21	0.01571
mmu-miR-29b	−6.33	0.01723
mmu-miR-29c	−4.38	0.01636
mmu-miR-301a	−4.21	0.04486
mmu-miR-30a	−3.89	0.03876
mmu-miR-30e	−3.37	0.03622
mmu-miR-324-5p	−4.63	0.04475
mmu-miR-328	−3.75	0.04278
mmu-miR-329	−4.79	0.01774
mmu-miR-330	−8.50	0.00381
mmu-miR-340-3p	−4.96	0.02453
mmu-miR-340-5p	−3.83	0.03845
mmu-miR-369-3p	−17.18	0.04086
mmu-miR-369-5p	−3.64	0.02940
mmu-miR-491	−4.04	0.02354
mmu-miR-497	−6.93	0.00462
mmu-miR-501-3p	−3.23	0.00474
mmu-miR-532-5p	−4.21	0.04129
mmu-miR-544	−3.89	0.01407
mmu-miR-652	−5.73	0.02815
mmu-miR-7a	−8.35	0.00062
mmu-miR-9	−3.88	0.01623
mmu-miR-92a	−4.34	0.01318
mmu-miR-93	−6.98	0.01638
mmu-miR-99b	−3.56	0.02227

**Figure 2:**
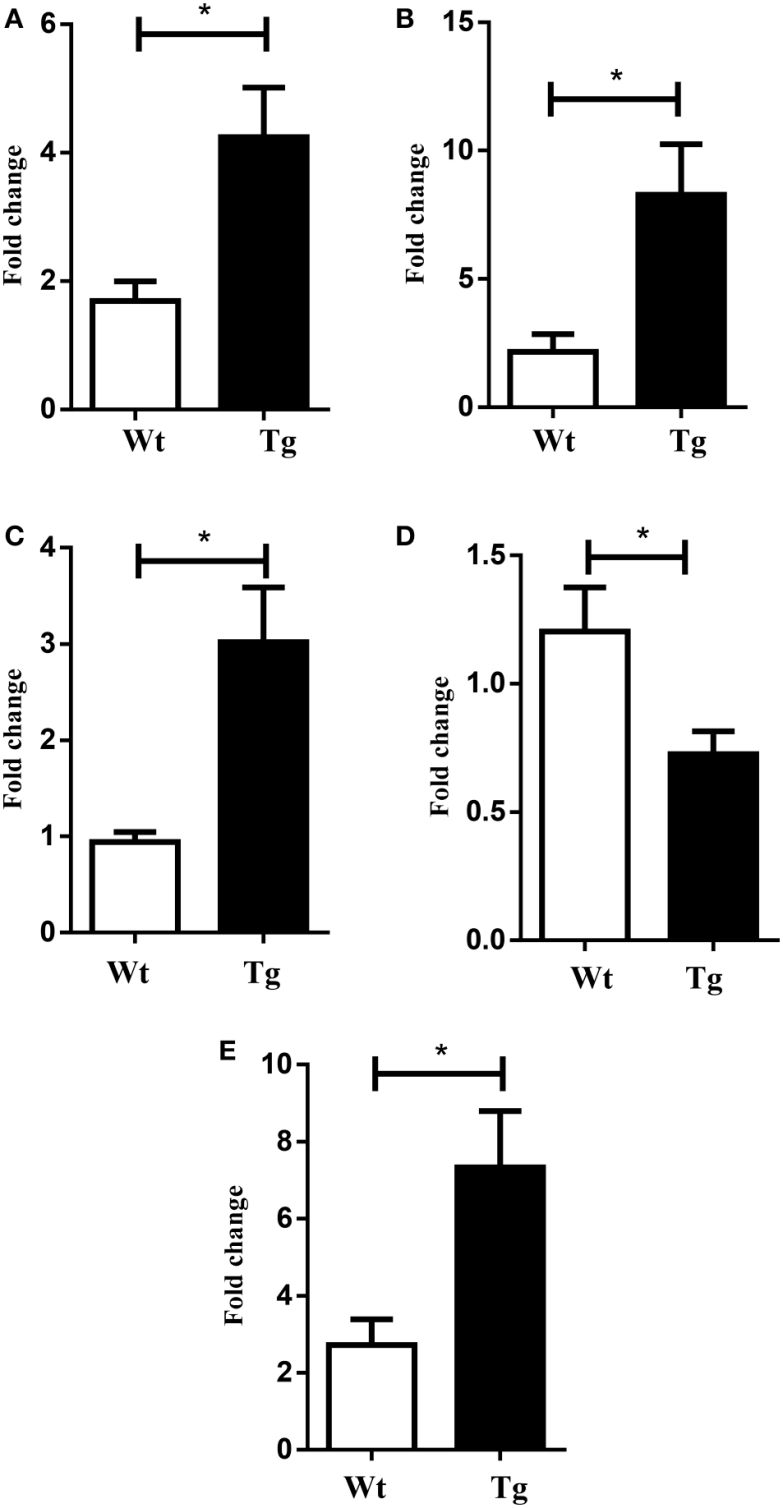
**Validation of selected mRNAs by Real-time PCR**. Graphs **(A)** Pmp 22 **(B)** Prx **(C)** Mpz **(D)** Opalin **(E)** Egr2. White bar refers to wild type (Wt) and black bar to MLC/SOD1^G93A^ (Tg). Values are expressed as mean ± SEM. ^*^p < 0.05 compared to Wt (*n* = 4 for each group).

The microarray data, related to the expression of mRNA in the spinal cord of both wild type and MLC/SOD1^G93A^ transgenic mice, were deposited in the GEO database (GSE69582) and the list of the selected significantly up-regulated and down-regulated genes (*p* < 0.05; *FC* > 1.5) is shown in Tables 2, 3 respectively. Among these genes, nine up-regulated transcripts are involved in the myelination process such as Peripheral myelin protein 22 (Pmp22), Myelin protein zero (Mpz), Periaxin (Prx), the Early growth response 2 (Egr2) genes, Desert hedgehog (Dhh), or encode for extracellular matrix molecules such as Matrilin 2 (Matn2), Gliomedin (Gldn), Claudin 19 (Cldn19), and Smoothelin (Smtn). Other relevant modulated genes include glycoproteins, such as Osteoglycin (Ogn) and dystroglicans, such as Dystrophin related protein 2 (Drp2), associated with the myelination process. The Mpz, Pmp22, Egr2, Prx mRNAs were selected for validation by qRT-PCR analysis and results are shown in Figure 2. Opalin, another gene associated to myelin structures, was down-regulated in the spinal cord of MLC/SOD1^G93A^ transgenic mice, compared to wild type littermates (Table 3). Opalin is expressed specifically in late stage of oligodendrocyte differentiation and has been shown to be dramatically reduced in a hypomyelination mouse model (Jiang et al., 2013; Tsutsumi et al., 2014).

**Table 2:** **List of significantly up-regulated genes in MLC/SOD1^G93A^ spinal cord**.

**Gene symbol**	**Gene name**	**Fold change**	***P*-value**
Arhgap19	Rho GTPase activating protein 19	+2.14	0.018071
Arl4d	ADP-ribosylation factor-like 4D	+1.51	0.026430
Art3	ADP-ribosyltransferase 3	+1.63	0.003088
Ccdc37	Coiled-coil domain containing 37	+1.67	0.013866
Chrdl1	Chordin-like 1	+1.96	0.010071
Cldn19	Claudin 19	+1.74	0.014558
Ddn	Dendrin	+1.51	0.022150
Dhh	Desert hedgehog	+1.86	0.021201
Drp2	Dystrophin related protein 2	+1.79	0.012309
Egr2	Early growth response 2	+1.70	0.022549
Gas2l3	Growth arrest-specific 2 like 3	+2.05	0.030748
Gimap1	GTPase, IMAP family member 1	+1.59	0.047143
Gldn	Gliomedin	+1.77	0.010186
Gulp1	GULP, engulfment adaptor	+1.63	0.006082
Hif3a	Hypoxia inducible factor 3, alpha subunit	+1.57	0.014288
Hist2h2ab	Histone cluster 2, H2ab	+1.56	0.002544
Hoxc13	Homeobox C13	+1.60	0.045743
Hoxd10	Homeobox D10	+2.13	0.043521
Il16	Interleukin 16	+1.64	0.011144
Inmt	Indolethylamine N-methyltransferase	+1.91	0.016648
Krtap4-2	Keratin associated protein 4-2	+1.54	0.005207
Matn2	Matrilin 2	+1.74	0.006453
Mertk	c-mer proto-oncogene tyrosine kinase	+1.80	0.003151
Mme	Membrane metallo endopeptidase	+1.58	0.018812
Mpz	Myelin protein zero	+1.64	0.049548
Nr4a2	Nuclear receptor subfamily 4, group A, member 2	+1.52	0.013349
Ogn	Osteoglycin	+2.02	0.041760
Olfr102, Olfr100	Olfactory receptor 102,100	+2.07	0.001166
Olfr128	Olfactory receptor 128	+1.78	0.025072
Olfr1283	Olfactory receptor 1283	+1.52	0.030795
Plekha4	Pleckstrin homology domain containing, family A	+2.01	0.020446
Pmp22	Peripheral myelin protein 22	+1.57	0.022466
Prrg4	Proline rich Gla (G-carboxyglutamic acid) 4	+1.66	0.000427
Prx	Periaxin	+1.98	0.019790
Rabggtb,	RAB geranylgeranyl transferase, b subunit	+1.77	0.041637
Rdh13	Retinol dehydrogenase 13 (all-trans and 9-cis)	+2.30	0.034523
Rhox2h	Reproductive homeobox 2H	+1.72	0.044442
Rpl31-ps4	Ribosomal protein L31, pseudogene 4	+1.61	0.004327
Sbp	Spermine binding protein	+1.60	0.000512
Sbspon	somatomedin B and thrombospondin	+1.66	0.009017
Slc36a2	Solute carrier family 36, member 2	+2.24	0.004210
Smco3	Single-pass memb. protein coiled-coil domains 3	+1.52	0.004448
Smtn	Smoothelin	+1.55	0.028145
Snhg3	Small nucleolar RNA host gene 3	+2.65	0.016377
Snora17	Small nucleolar RNA, H/ACA box 17,	+2.74	0.027109
Snora73b,	Small nucleolar RNA, H/ACA box 73b,	+2.23	0.025905
Sostdc1	Sclerostin domain containing 1	+1.68	0.007660
Tdpoz1	TD and POZ domain containing 1	+1.68	0.010976
Tgfbi	Transforming growth factor, beta induced	+1.83	0.027616
Th	Tyrosine hydroxylase	+1.72	0.025458
Vmn1r181	Vomeronasal 1 receptor 181	+1.51	0.042949
Xlr4b	X-linked lymphocyte-regulated 4B	+1.88	0.005473
Zbtb16	Zinc finger and BTB domain containing 16	+1.53	0.018846
Zfp37	Zinc finger protein 37	+1.54	0.000034

**Table 3:** **List of significantly down-regulated genes in MLC/SOD1^G93A^ spinal cord**.

**Gene symbol**	**Gene name**	**Fold change**	***P*-value**
Akr1c20	Aldo-keto reductase family 1, member C	−1.64	0.031454
Aplnr	Apelin receptor	−1.78	0.020211
C3, LOC100048759	Complement component 3, complement C3-like	−1.60	0.044240
Cyp2j12	Cytochrome P450, family 2, subfamily j, polypeptide 12	−1.58	0.018190
Enpp6	Ectonucleotide pyrophosphatase/phosphodiesterase 6	−1.58	0.015427
Frmpd4	FERM and PDZ domain containing 4	−1.66	0.010645
Gbp4	Guanylate binding protein 4	−1.65	0.027557
Gm14257	Predicted gene 14257	−1.54	0.032704
Gm15246, BC022960	Predicted gene 15246, cDNA sequence BC022960	−1.84	0.029954
Gm3088	Predicted gene 3088	−1.61	0.048993
Klk6	Kallikrein related-peptidase 6	−1.70	0.005161
mt-Tt	Mitochondrially encoded tRNA threonine	−1.52	0.036295
Nkx2-9	NK2 transcription factor related, locus 9 (Drosophila)	−1.51	0.007001
Olfr611	Olfactory receptor 611	−4.75	0.000275
Olfr727	Olfactory receptor 727	−1.51	0.043050
Opalin	Oligodendrocytic myelin paranodal and inner loop prot.	−1.75	0.000707
Pi15	Peptidase inhibitor 15	−1.52	0.020502
Prok2	Prokineticin 2	−1.54	0.040329
Prps2	Phosphoribosyl pyrophosphate synthetase 2	−1.60	0.034506
Serpina1c	Serine peptidase inhibitor, clade A, member 1C	−1.66	0.024381
Vmn1r37	Vomeronasal 1 receptor 37	−1.62	0.023405
Vmn2r71	Vomeronasal 2, receptor 71	−1.70	0.033296
Vnn1	Vanin 1	−1.65	0.010154

**Figure 3:**
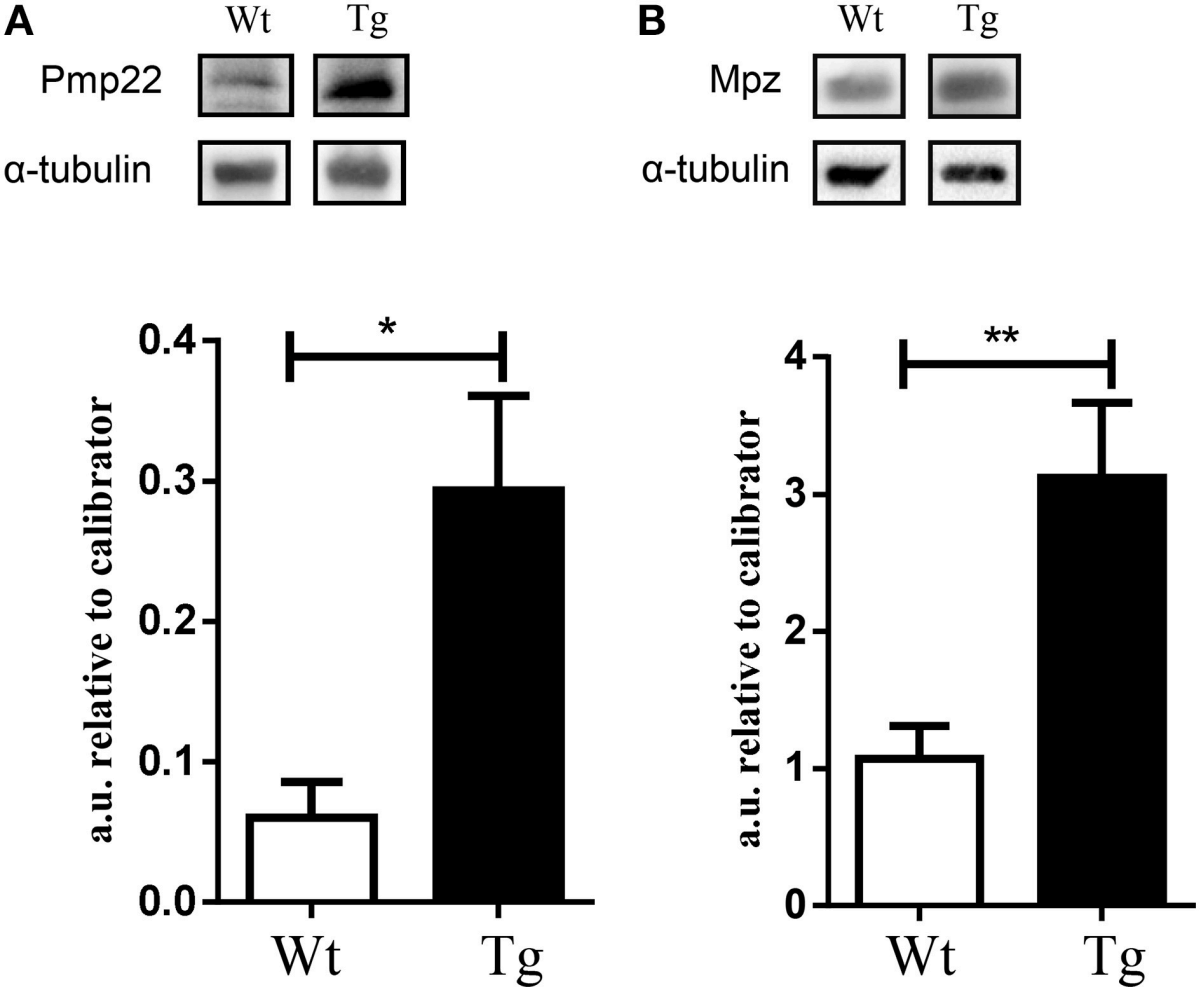
**Validation of selected factors by western blot analysis**. Graphs and representative western blot for **(A)** Pmp 22 and **(B)** Mpz. White bar refers to wild type (Wt) and black bar to MLC/SOD1^G93A^ (Tg). Values are expressed as mean ± SEM. ^*^p < 0.05; ^**^p < 0.005 compared to Wt (*n* = 5 for each group).

In order to show changes in protein expression, as a result of altered microRNA/mRNA expression, we performed western blot analysis for Pmp22 and Mpz expression. We revealed a significant up-regulation of Pmp22 and Mpz proteins in the spinal cord of MLC/SOD1^G93A^ compared to wild type littermates (Figure 3), further supporting the evidence that these factors are targets of specific microRNAs that control the denervation and reinnervation processes.

Taken together these results suggest that the muscle specific expression of the mutated isoform of the human SOD1 gene alters the expression of relevant markers of myelination in the ventral root of the lumbar spinal cord of transgenic mice.

### Functional annotation of the identified genes

**Figure 4:**
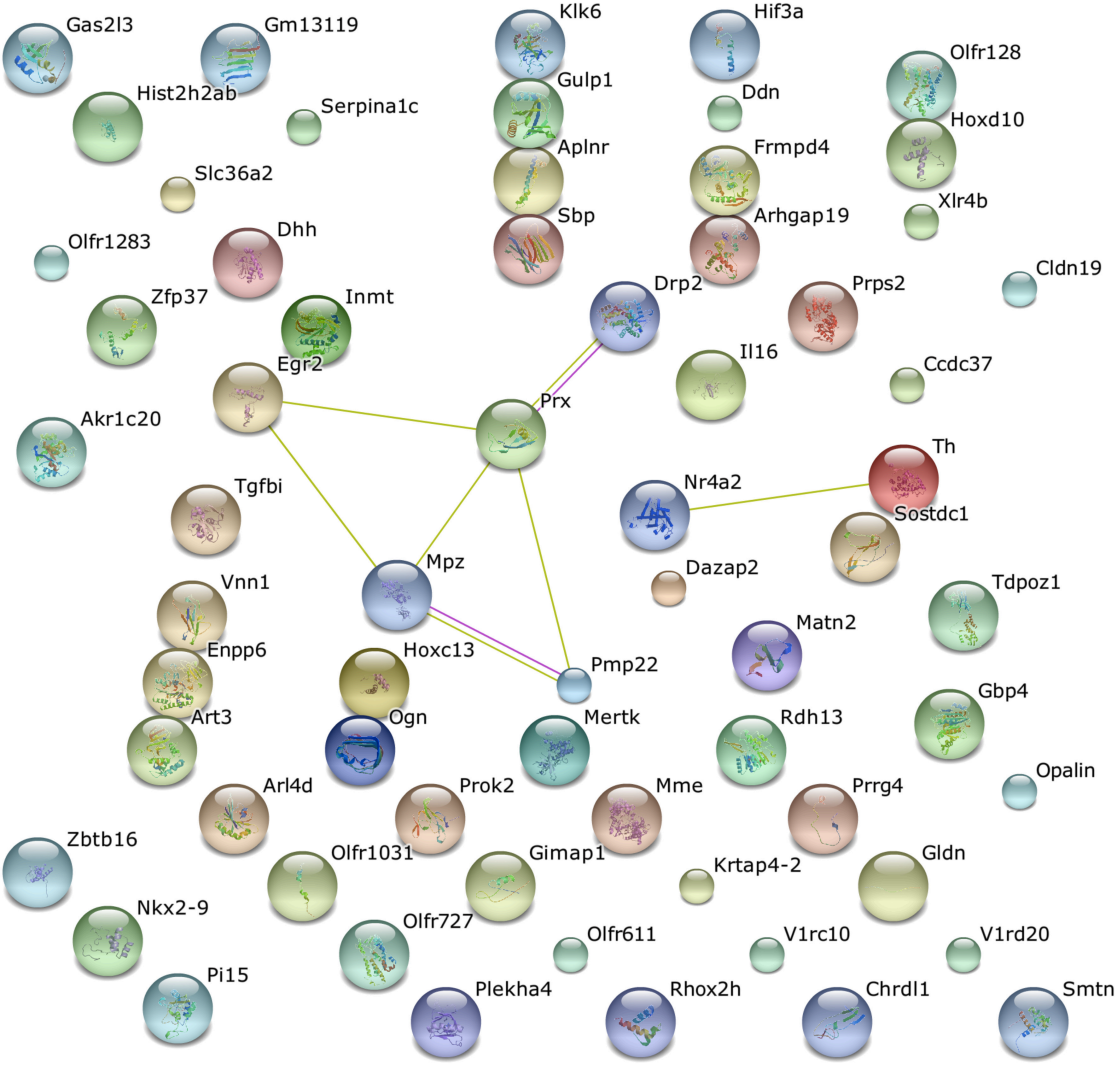
**STRING analysis of pathway enrichment and interaction in the altered mRNAs of the MLC/SOD1^G93A^ transgenic mice**. Protein products of five modulated mRNAs (Drp2, Prx, Egr2, Mpz, and Pmp22) were involved in eight interactions.

Functional annotation of differentially expressed genes was performed using the DAVID functional annotation tool, an integrated biological knowledge base and analytic tools aimed at systematically extracting biological meaning from large gene and protein lists (Huang da et al., 2009). Similar annotation contents were clustered into annotation group with biological meanings. The annotation results revealed that 2.6% of the gene list examined was consistent with OMIM disease categories, in particular 5 of 17 genes extracted were related to Charcot-Marie-Tooth disease. On the other hand, the gene ontology analysis revealed that 39.3% of all gene list are significantly involved in biological processes category, 36.7% are part of cell components, and 39.8% are associated with molecular function categories. The nine interesting genes mentioned above were spread out in all the categories examined. The functional annotation clustering was done using the default parameters, *Mus musculus* as background and classification stringency was set as medium. Table 4 shows that the significantly modulated genes were involved in 5 functional clusters containing functional annotation for extracellular region, neuron development and differentiation, axonogenesis, and neurological system process. A further functional analysis was performed using STRING, a web base tool to explore potential protein-protein interaction, confirming the interactions among Pmp22, Mpz, Prx, and Egr2 proteins (Figure 4).

**Table 4:** **Functional annotation clustering of up-regulated mRNAs**.

**Annotation cluster 1**	**Enrichment score: 1.85**	
**Category**	**Count**	***P*-value**
GOTERM_CC_FAT extracellular region	15	2.7E-3
GOTERM_CC_FAT extracellular region part	8	2.4E-2
GOTERM_CC_FAT extracellular space	6	4.2E-2
**Annotation cluster 3**	**Enrichment score: 0.95**	
GOTERM_BP_FAT neuron development	5	1.4E-2
GOTERM_MF_FAT transcription factor activity	7	3.8E-2
GOTERM_BP_FAT neuron differentiation	5	4.0E-2
**Annotation cluster 4**	**Enrichment score: 0.87**	
GOTERM_CC_FAT extracellular region part	8	2.4E-2
**Annotation cluster 5**	**Enrichment score: 0.8**	
GOTERM_BP_FAT locomotory behavior	5	7.3E-3
GOTERM_BP_FAT behavior	6	9.6E-3
GOTERM_BP_FAT pattern specification process	5	1.3E-2
GOTERM_BP_FAT neuron development	5	1.4E-2
GOTERM_BP_FAT response to endogenous stimulus	4	2.2E-2
GOTERM_BP_FAT regionalization	4	3.2E-2
GOTERM_MF_FAT transcription factor activity	7	3.8E-2
GOTERM_BP_FAT neuron differentiation	5	4.0E-2
**Annotation cluster 7**	**Enrichment score: 0.53**	
GOTERM_BP_FAT neurological system process	11	3.9E-2

*Annotation cluster means a group of terms having similar biological functions. Enrichment score is the rank of the annotation cluster biological significance. Count column refers to number of genes involved in the same category*.

### Correlation of expression profile between miRNAs and mRNA

miRNAs are key regulators of all important biological processes and the level at which they act on coding mRNA (transcriptional, translational, etc.) is still debated; however gene repression by microRNAs at the level of mRNA translation is the most frequently reported mechanism (Morozova et al., 2012). Therefore, we further performed a systematic analysis on the interaction between significantly modulated miRNAs and mRNAs considering only miRNAs present in our list with an inverse mRNAs expression correlation. The analysis was performed using MiRwalk tool a comprehensive database that provides information on miRNA produced by 8 established miRNA prediction programs on 3′ UTRs of all known genes of mouse such as RNA22, miRanda, miRDB, TargetScan. Secondly, we ordered mRNAs on the basis of the number of putative targeting miRNAs and we observed that 36 genes were targeted from at least 1 to maximum 30 significantly modulated miRNAs; conversely 54 listed miRNAs targeted at least one to maximum of 11 significantly modulated genes; interestingly the most intriguing cross interaction involved genes and miRNAs of the myelination process (Table 5).

**Table 5:** **List of selected genes and corresponding regulated miRNAs**.

**Gene**	**miRNA**	**miRNA**	**Gene**
Egr2	mmu-miR-93	mmu-miR-1	Cldn19
	mmu-miR-17		DrP2
	mmu-miR-20b		Mpz
Mpz	mmu-miR-1		Nr4a2
	mmu-miR-129-3p		Ogn
	mmu-miR-133a		Prx
	mmu-miR-133b		Drp2
	mmu-miR-192	mmu-miR-9	Dhh
	mmu-miR-30a		Mme
	mmu-miR-30e		Pmp22
	mmu-miR-329		Prx
	mmu-miR-491		Rdh13
Pmp22	mmu-miR-26b		Tgfbi
	mmu-miR-29a	mmu-miR-15	Cldn19
	mmu-miR-29b		Gldn
	mmu-miR-29c		Prx
	mmu-miR-330		Rdh13
	mmu-miR-340-3p		
	mmu-miR-501-3p	mmu-miR-29	Drp2
	mmu-miR-544		Gldn
	mmu-miR-9		Pmp22
Prx	mmu-miR-1	mmu-miR-133 a–b	Cldn19
	mmu-miR-106a		Mpz
	mmu-miR-10a		Rdh13
	mmu-miR-10b		
	mmu-miR-128		
	mmu-miR-129-3p		
	mmu-miR-15a		
	mmu-miR-15b		
	mmu-miR-17		
	mmu-miR-188-5p		
	mmu-miR-206		
	mmu-miR-20b		
	mmu-miR-328		
	mmu-miR-330		
	mmu-miR-491		
	mmu-miR-497		
	mmu-miR-7a		
	mmu-miR-9		
	mmu-miR-93		

*Schematic representation of the inversely correlated miRNAs and predicted targets based on Mirwalk analysis*.

This finding clearly suggests that muscle specific overexpression of the SOD1 mutant gene induces the modulation of miRNAs and putative targeted-mRNAs involved in the myelination process in the spinal cord of MLC/SOD1^G93A^ transgenic mice.

### Mapping miRNAs to signaling pathways

DIANA-mirPath is a web-based computational tool to identify KEGG signaling pathways regulated by miRNAs (see Materials and Methods section). The software compares each set of miRNA targets with all-known KEGG pathway and interestingly we observed high significance for axon guidance, glutamatergic synapse, and amyotrophic lateral sclerosis (ALS), meaning that these pathways are likely to be controlled by the altered miRNAs (Table 6).

**Table 6:** **Signaling pathways predicted to be regulated by miRNAs**.

**KEGG Pathway**	***P*-value**	**Number of miRNA**
PI3K-Akt signaling pathway	5.368871e-57	48
MAPK signaling pathway	2.427724e-54	46
Focal adhesion	9.558692e-36	44
Wnt signaling pathway	1.247526e-33	42
Axon guidance	3.168282e-33	42
Ubiquitin mediated proteolysis	3.031889e-27	37
Neurotrophin signaling pathway	3.031889e-27	42
Insulin signaling pathway	1.208302e-26	45
ErbB signaling pathway,	6.307645e-22	46
Transcriptional misregulation in cancer	9.134568e-22	45
Protein processing in endoplasmic reticulum	1.448874e-21	40
TGF-beta signaling pathway	4.891741e-21	36
HIF-1 signaling pathway	2.055494e-20	43
Adherens junction	4.208319e-16	36
Jak-STAT signaling pathway	2.747301e-15	39
mTOR signaling pathway	1.21693e-14	38
VEGF signaling pathway	1.531543e-13	38
Glutamatergic synapse	1.114872e-10	38
Amyotrophic lateral sclerosis (ALS)	7.053373e-10	34
Notch signaling pathway	9.441105e-09	29
Toll-like receptor signaling pathway	3.1133e-08	35
Circadian rhythm	8.717738e-08	32
Dorso-ventral axis formation	3.707892e-06	28
Adipocytokine signaling pathway	8.449379e-06	33
Dopaminergic synapse	1.421481e-05	38
Dilated cardiomyopathy	6.37123e-05	35
Cholinergic synapse	0.0001426234	36
Protein digestion and absorption	0.0004322993	35
mRNA surveillance pathway	0.0005316409	36
Cytokine-cytokine receptor interaction	0.00200525	43
Calcium signaling pathway	0.03407104	42
D-Glutamine and D-glutamate metabolism	0.03934887	5
Carbohydrate digestion and absorption	0.0460262	20

### Muscle specific expression of SOD1 mutant gene induces hypomyelination in the mice sciatic nerve

**Figure 5:**
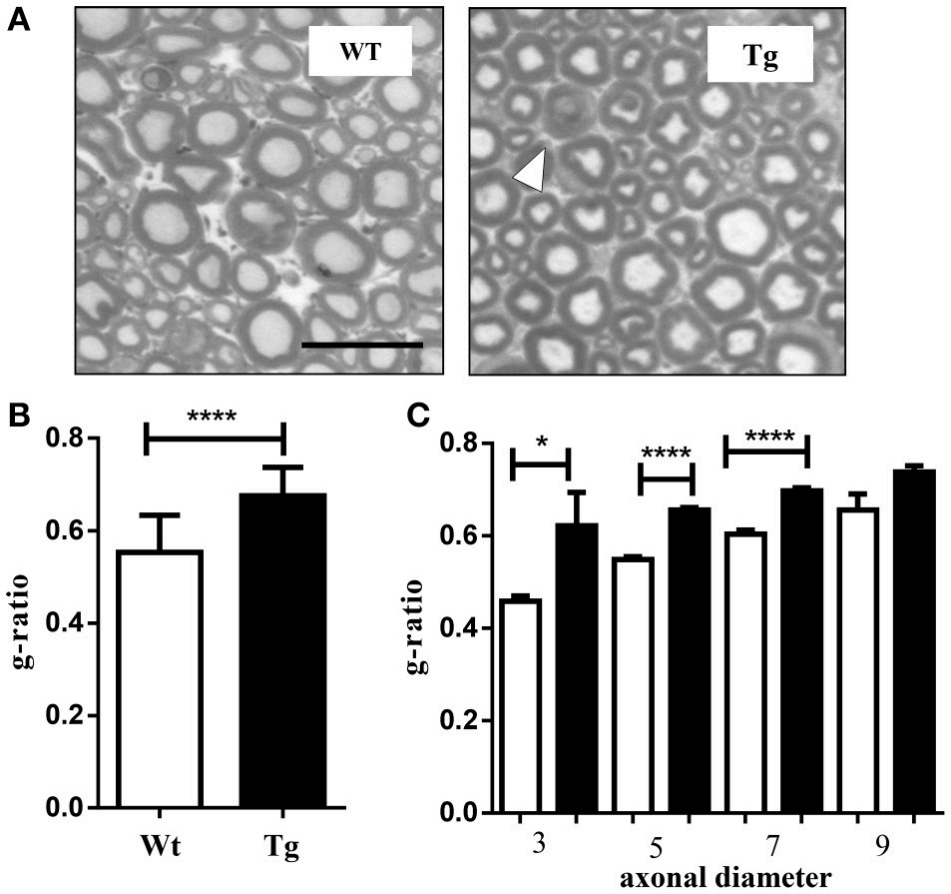
**Hypomyelination in the sciatic nerve of MLC/SOD1^G93A^ mice**. **(A)** Photographs showing the mouse sciatic nerve strained with toluidine blue. White arrowhead indicates onion bulb structure (40X magnification) Scale bar = 20 μm. **(B)** The average g-ratios. **(C)** The average g-ratio for each axonal diameter (1–3, 3–5, 5–7, >7 μm). Values are expressed as mean ± SEM fibers. ^*^p < 0.05; ^****^p < 0.0001 compared to wild type (Wt).

Different reports indicate that ALS might be associated with motor nerve fiber (Echaniz-Laguna et al., 2006; Rajabally and Jacob, 2008; Ahdab et al., 2013). Previously, we demonstrated that the peripheral nerve was severely compromised in the global SOD1^G93A^ mutant mice, which displayed loss of Schmidt-Lantermann incisures, a disproportionately thick myelin sheath, abundant double onion bulb structures, and increased demyelinated axons (Dobrowolny et al., 2008a). To assess whether muscle specific expression of SOD1 mutant gene induces a peripheral alteration of axon myelination we calculated the ratio of axon diameter to total fiber diameter (g-ratio) (Michailov et al., 2004). The Figure 5A shows the cross-sections of wild type and MLC/SOD1^G93A^ sciatic nerves stained with toluidine blue. The histological analysis of the peripheral nerve of the MLC/SOD1^G93A^ displayed the presence of double onion bulb structures. Moreover, the g-ratio in the transgenic sciatic nerves was significantly increased (Figure 5B) independently of the axonal diameter (Figure 5C), indicating that muscle specific expression of SOD1 mutant gene induces hypomyelination in the sciatic nerve of transgenic mice.

## Discussion

The functional interplay between muscle and nerve is crucial for the survival and function of both tissues throughout life and ALS represents one of the best examples of impaired interplay between the two tissues (Musarò, 2013). Although, there have been significant advances in our understanding of the biology of ALS, no consensus has emerged as to which cells, tissues and pathways are directly affected by mutant SOD1. Motor neurons degeneration and muscle atrophy are the major pathological processes associated with ALS consistent with the role of nerve activity in muscle homeostasis and remodeling (Munson et al., 1997; Pette, 2001; Schiaffino et al., 2007). However, other cells may be involved in the pathogenesis of ALS and alteration in skeletal muscle homeostasis might represent one of the critical mediators of motor neuron degeneration. This hypothesis has been recently investigated by different groups, and we recently demonstrated that muscle selective expression of SOD1 mutation causes pathological alterations in skeletal muscle and induces pre-symptomatic sign of ALS at the level of spinal cord (Dobrowolny et al., 2008b).

In the present study, we extended our previous work and analyzed whether muscle-specific expression of SOD1^G93A^ is able to affect the expression of non-coding RNA and mRNA in the spinal cord of MLC/SOD1^G93A^ transgenic mice, compared with wild type littermates. Our findings are in agreement with previous studies showing that skeletal muscle is also a source of signals that influence neuron survival, axonal growth and maintenance of synaptic connections (Funakoshi et al., 1995; Dobrowolny et al., 2005). In the absence of trophic support, muscle can have a negative impact on the nervous system, and therefore, can contribute to the alteration in the functional connection between muscle and nerve.

The major findings of our study indicate that muscle-specific expression of SOD1^G93A^ can modulate relevant molecules of the genetic and epigenetic circuitry of the myelin homeostasis in the spinal cord of transgenic mice. In particular, we found down regulation of mir-1, mir-330, mir-29, mir-133, and mir-9 family members, whose dysregulation can have profound effects on neuronal physiology and pathology, including Huntington, Alzheimer, and Parkinson diseases (Saito and Saito, 2012). Although, it is known that deregulation of specific miRNA-dependent regulatory circuitries correlates with the initiation and progression of several neurological disorders, the underlying mechanisms of these phenomena are still not fully understood.

Here we revealed the alteration of specific microRNAs and potential mRNA targets, which might be part of the short circuit that disrupts the communication between skeletal muscle and nerve and that might be part of the so called dying back phenomenon (Dadon-Nachum et al., 2011). In particular, among transcripts that are potential targets of the altered miRNAs, we revealed the presence of relevant markers of myelin homeostasis that are modulated by muscle restricted expression of SOD1 mutation inducing hypomyelination of the peripheral nerve. The up-regulation of Pmp22 and Mpz proteins in the spinal cord of MLC/SOD1^G93A^ paralleled that of mRNA expression and supports the evidence that these factors are molecular targets of microRNAs, such as miR-1, miR-9, miR-133, and miR-330, that resulted differently modulated in the spinal cord of MLC/SOD1^G93A^ mice compared to wild type littermates.

It has been demonstrated that Myelin protein zero is up-regulated in oligodendroglia following axonal injury (da Silva et al., 2015) and Periaxin is expressed by myelinating Schwann cells in association with the dystroglycan complex through Drp2 (Sherman et al., 2012). Moreover, Pmp22 exhibits a dose dependent function as both overexpression and deletion result in hereditary neuropathy (Han et al., 2013). Dhh is essential for the structural and functional integrity of the peripheral nerve (Sharghi-Namini et al., 2006) and Osteglycine, Claudin -19 and Smoothelin together with Pmp22 and Prx are associated with reduced myelination of the spinal cord in the hypogravity motor syndrome (Chelyshev et al., 2014). Moreover, mutations in Mpz, Pmp22, Egr2, and Prx are associated with Charcot–Marie–Tooth neuropathy in which the over expression of these genes leads to defective myelination processes.

Recently, some specific miRNAs, such as miR-9, miR-23, and miR-29a, were found to participate in the regulation of oligodendrocyte differentiation and myelin maintenance, as well as in the pathogenesis of demyelination-related diseases. These miRNAs control their target mRNAs and are involved in the pathogenesis of demyelination-related diseases (Lau et al., 2008; Verrier et al., 2009; Li and Yao, 2012).

Regarding the mRNA-miRNA interplay, most of the putative interactions proposed here have not been previously described and deserve further investigation. Interestingly, the functional interaction of miR-9 with Peripheral myelin protein 22 (Pmp22) mRNA has been already demonstrated (Lau et al., 2008). In particular, miR-9 is down-regulated during oligodendrocyte differentiation and its expression level inversely correlates with the expression of its targets Pmp22. Moreover, it has been described that the inhibition of endogenous miR-29 overrides the miRNA-mediated repression of Pmp22 cultured Schwann cells during both development and post-crush injury (Verrier et al., 2009). Therefore, these data suggest that myelin gene expression is regulated by miRNAs.

Here we observed that the muscle specific expression of the SOD1 mutant gene induces the deregulation of both mir-9 and 29 together with myelin alteration in the sciatic nerve. These data have been validated by histological analysis of the sciatic nerve that shows a reduction in the myelin sheath in the MLC/SOD1^G93A^ mice model. In this regard it is interesting to note that the classical oligodendrocyte markers were down-regulated (considering a fold change < 1.5 olig2 *FC* = −1.3 *p* = 0.02; Nkx2-2 FC = −1.36; *p* = 0.008; Mag FC = −1.39, *p* = 0.01). Therefore the reduction in oligodendrogenesis could be correlated with the hypomyelination in the MLC/SOD1^G93A^ mouse model.

In the spinal cord of SOD1^G93A^ transgenic mice that ubiquitously express the mutant SOD1 gene, the miRNA-9 expression is up-regulated (Zhou et al., 2013). In this model there is extensive degeneration of gray matter oligodendrocytes in the spinal cord prior to disease onset; actually new oligodendrocytes were formed but they failed to mature, resulting in progressive demyelination (Kang et al., 2013). The difference in the expression levels of miRNA-9 between the global SOD1^G93A^ and MLC/SOD1^G93A^ mice is likely due to SOD1^G93A^ gene ubiquitously expressed in all tissues, including muscle, motor neurons and glia. This might generate a synergistic toxic effect, leading to a more severe phenotype.

Although, most of the miRNA and mRNA inverted correlations proposed here have not been yet validated, the elevated number of potential interactions strongly points toward a role of skeletal muscle in myelin homeostasis. Nevertheless, although these results point toward some mechanisms of action of muscle specific expression of SOD1^G93A^ at the level of spinal cord, the elevated number of potential targets of the analyzed miRNAs make these mechanisms only mere suggestions. Additional studies will define which cell types, at the levels of spinal cord of MLC/SOD1^G93A^ mice, specifically exhibit changes in microRNAs and mRNAs.

Overall our study provides additional insights into the effects of muscle selective expression of SOD1^G93A^ on nerve homeostasis and reveals the potential miRNA and mRNA signature associated with the dying back phenomenon.

## Conflict of interest statement

The authors declare that the research was conducted in the absence of any commercial or financial relationships that could be construed as a potential conflict of interest.

## Abbreviations

ALS: Amyotrophic Lateral Sclerosis
ANOVA: ANalysis Of VAriance
Cldn19: Claudin 19
DAVID: Database for Annotation Visualization Integrated Discovery
Dhh: Desert hedgehog
DIANA: Dna Intelligence ANAlysis
dmax: maximum axonal diameter
Dmax: maximum fiber diameter
Dmin: minimum fiber diameter
dmin: minimum axonal diameter
Drp2: Dystrophin related protein 2
Egr2: Early growth response 2
FC: Fold Change
FVB: Friend leukemia virus B strain
GEO database: Gene Expression Omnibus database
Gldn: Gliomedin
GO: Gene Ontology
KEGG: Kyoto Encyclopedia of Genes and Genomes
Mag: Myelin-associated glycoprotein
Matn2: Matrilin 2
mIGF-1: muscle specific isoform of the Insulin Growth Factor-1
miRNA, miR: microRNA
miRPath: miRNA pathway
MLC: Myosin Light Chain
Mpz: Myelin protein zero
Nkx2-2: NK2 homeobox 2
Ogn: Osteoglycin
OMIM: Online Mendelian Inheritance in Man
Pmp22: Peripheral myelin protein 22
Prx: Periaxin
SDS: Sodium Dodecyl Sulfate
Smtn: Smoothelin
SOD1: Superoxide dismutase 1
STRING: Search Tool for the Retrieval of Interacting Genes/Proteins
TAC: Transcriptome Analysis Console
UTR: Untranslated region.
